# Consecutive drilling combined with phaco chop for full thickness segmentation of very hard nucleus in coaxial microincisional cataract surgery

**DOI:** 10.1186/s12886-019-1033-1

**Published:** 2019-01-16

**Authors:** Ding Chen, Qunwu Tang, Fang Yu, Xueting Cai, Fan Lu

**Affiliations:** Eye Hospital, Wenzhou Medical University, 270 Xueyuan Road, Wenzhou, 325027 Zhejiang China

**Keywords:** Phacoemulsifcation, Hard nucleus, Microincision, Phaco chop, Phaco drill

## Abstract

**Background:**

The complete disassembly of nuclear is the most challenging step in hard cataract surgery through microincision. The classic phaco chop technique often does not succeed, resulting in incomplete nuclear segmentation. The authors describe a technique to improve the efficacy and safety of the initial chopping.

**Methods:**

The consecutive drilling combined with phaco chop technique was devised for very hard cataract through a microincision of 1.8–2.2 mm. 3–4 holes are consecutively drilled into the endonucleus with the phaco tip bevel down, at an angle of approximate 60 degrees and depth of approximately two-thirds of the lens thickness. The initial drilling approaches the capsulorhexis edge and the last drilling approaches the lens geometric center. The nucleus is deeply impaled with the last drilling and firmly engaged with high vacuum, and then chopped with chopper centripetally from the lens equator. The chopper and phaco tip are spread apart laterally after they approach at the center of the nucleus, to create a complete fracture across the entire nucleus. This technique has been adopted in 80 eyes of 65 patients with cataract harder than nuclear opalescence 5 on the Lens Opacities Classification System III scale or mature white cataract with a hard nucleus in the past 12 months.

**Results:**

In all cases, full thickness segmentation of the hard nuclear including the posterior plate was achieved with this consecutive drilling combined with phaco chop technique. Phacoemulsification and intracapsular implantation of intraocular lens was safely performed in each case. No intraoperative complication such as iris injury, anterior capsule tears, zonulysis or posterior capsule rupture with vitreous loss occurred during surgery. No postoperative complication such as fibrin formation, synechias, severe endothelial cell loss, or endophthalmitis was observed in any case at 6 months postoperatively.

**Conclusions:**

The technique is an efficient, safe, simple, and swift procedure for full-thickness nuclear segmentation, delivering advantage of microincisional phacoemulsifcation for hard cataract with few ocular complications.

**Electronic supplementary material:**

The online version of this article (10.1186/s12886-019-1033-1) contains supplementary material, which is available to authorized users.

## Background

Hard nuclear cataracts are commonly seen in many people, especially in China rural population. Due to the advances in phacoemulsifcation machines and surgical techniques, phacoemulsifcation of hard cataracts is more widely performed than extracapsular cataract extraction. However, it has always been a challenge to perform coaxial microincision phacoemulsifcation of cataracts harder than nuclear opalescence 5 on the Lens Opacities Classification System III [1]. Higher ultrasound energy, higher vacuum, and stronger forces for nuclear separation may be needed, which may increase the risk of complications such as corneal burn, endothelial trauma, zonular tear, and capsular rupture [2].

Successful surgery through microincision for hard cataract may depend on how well the surgeon divides the lens nucleus into multiple small fragments. The complete segmentation of the dense nuclear into 2 hemispheres becomes the crucial and most challenging step during the whole procedure. Phaco chop has been considered as one of the best techniques for dealing with hard cataracts [3]. However, phaco chop technique quite often does not succeed, resulting in incomplete nuclear segmentation and intact posterior plate. Surgeons may attempt to perform the subsequent chop in an unchopped region of the lens by rotating the lens but may exert extra stress to the zonular fiber and lens capsule.

We have devised an efficient technique “consecutive drilling combined with phaco chop” for full thickness segmentation of very hard nucleus in coaxial microincisional cataract surgery. This technique can fully crack the hard nuclear into two complete segments including the posterior plate with much ease and which is also free from high-intensity shock and strain.

## Methods

### Surgical technique

All coaxial microincisional cataract surgery (coaxial MICS) is performed under topical anesthesia through a clear corneal incision with a side port. The size of main incision can be either 1.8 mm or 2.2 mm depending on the coaxial MICS system adopted. Indocyanine green staining is necessary to visualize the anterior capsule when the cataract is white with a hard nucleus. Using the soft shell technique, the anterior chamber is filled with an ophthalmic viscosurgical device (OVD). The anterior capsule is opened using the continuous curvilinear capsulorhexis (CCC) with a diameter of approximately 6.0 mm. Hydrodissection is performed to separate the cortex from the capsule. For Stellaris Vision Enhancement System (Bausch & Lomb, Bridgewater, NJ, USA), a straight phaco tip with longitudinal ultrasound mode is used, while for Alcon Infiniti Intrepid phacoemulsification system (Alcon Laboratories, Inc., Fort Worth, TX, USA), a mini-flared 0.9 mm 30-degree Kelman tip with torsional mode is used. The phaco tip is introduced into the anterior chamber, and the superficial cortex and epinuclear plate are removed.

**Fig. 1:**
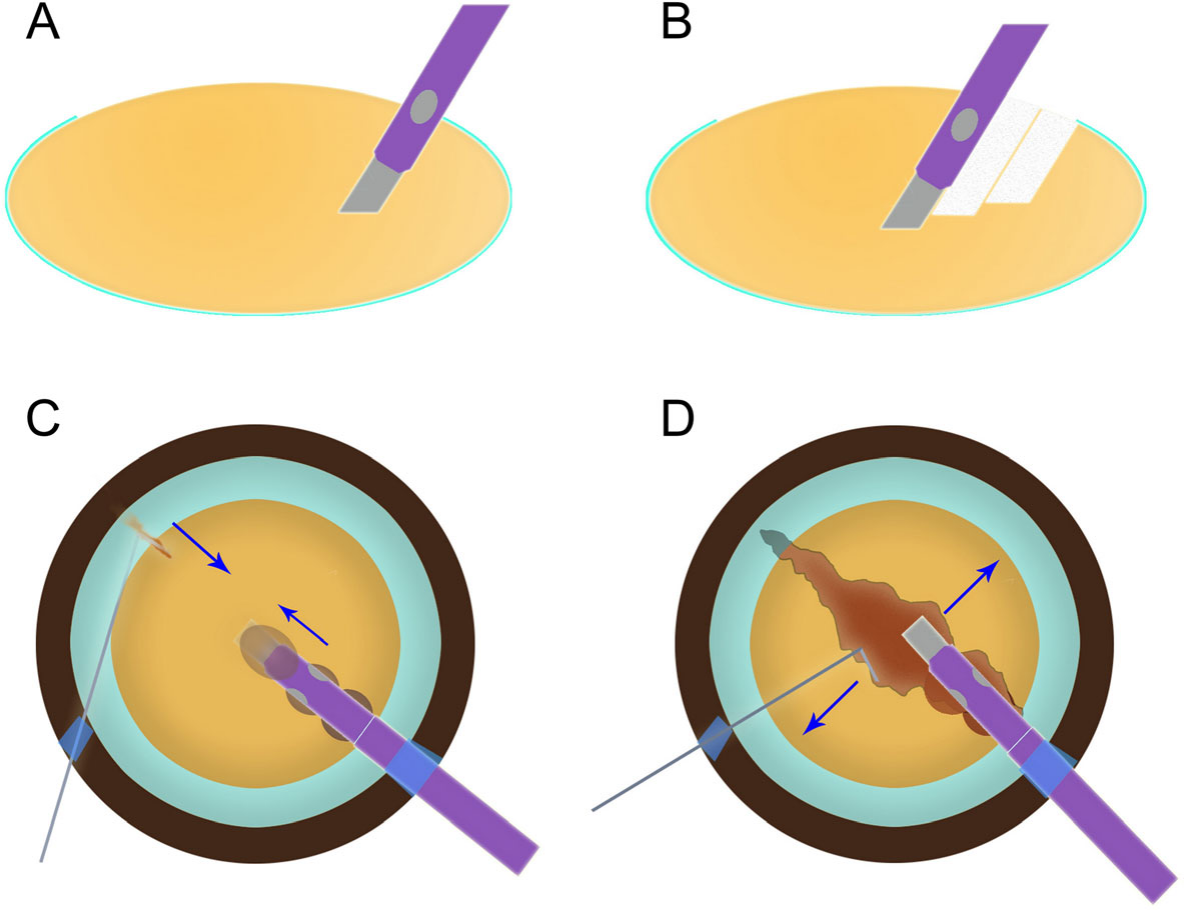
Illustration of the consecutive drilling combined with phaco chop technique. **a**: The initial drilling into the endonucleus is made from the capsulorhexis edge beneath the main incision with the phaco tip bevel down. The angle of drilling is approximate 60 degrees, and the depth is approximately two-thirds depth of the anteroposterior lens thickness. **b**: After consecutive 3–4 drillings towards the center of the nucleus, a deep groove from the capsulorhexis edge to the center of the nuclear is created. **c**: The nucleus is deeply impaled with the last drilling and firmly engaged with high vacuum, the chopper is then inserted under the capsule and moved horizontally from the nucleus periphery toward the phaco tip to split the nucleus. **d**: Both the phaco tip and chopper are then spread apart laterally as they come very close to propagate a complete division across the full thickness of the entire nucleus


The next step is to perform consecutive drillings into the endonucleus with the phaco tip bevel down. The phacoemulsifcation machine settings used are vacuum 400 mmHg, aspiration flow rate 30 mL/min, phaco power 90%, and infusion bottle height 90 cm. The initial drilling is made from the capsulorhexis edge beneath the main incision, followed by the next consecutive 2–3 drillings towards the center of the nucleus. (Fig. 1a) The angle of drilling is approximate 60 degrees, and the depth is approximately two-thirds depth of the anteroposterior lens thickness (Fig. 1b), which can be measured by placing the phaco tip with a known diameter. To maximize a deeper purchase, the silicone sleeve is retracted to expose the phaco tip at the length of approximate 3 mm. A deep groove from the capsulorhexis edge to the center of the nuclear is therefore created by the series of drilling. At the last drilling, the nucleus is deeply impaled and firmly engaged with high vacuum. Then the chopper is inserted under the capsule through the side-port and placed opposite the main incision at the edge of the nucleus. Simultaneously, the chopper is moved from the nucleus periphery toward the phaco tip horizontally to split the nucleus (Fig. 1c) Both the phaco tip and chopper are then spread apart laterally as they come very close to divide the nucleus into halves. When leathery posterior strands kept nuclear fragments attached to each other, the chopper and phaco tip are reposition at the bottom of the crack to break the strands and propagate a complete division across the full thickness of the entire nucleus. (Fig. 1d) The phacoemulsification tip is impaled in half the nucleus in situ without rotating the nucleus, and the chopper is used to break this half into 2 or more smaller fragments, which are then emulsified and aspirated in the capsular bag. The procedure is repeated in the other half of the nucleus in the same manner and the fragmentary nucleus is rotated when necessary. After the nuclear phacoemulsification is completed, the irrigation/aspiration tip is used to clean residual cortical material, followed by insertion and in-the-bag placement of the intraocular lens. The OVD is removed, and the corneal incisions are water sealed. Two cases with the technique on two different phacoemulsification systems were demonstrated in videos (Additional files 1 and 2: Video 1&2).

Additional file 1:Video 1. shows the application of consecutive drilling combined with phaco chop technique on Stellaris Vision Enhancement System (Bausch & Lomb, Bridgewater, NJ, USA), in which a straight phaco tip with longitudinal ultrasound mode is used through a microincision of 1.8 mm. After the superficial cortex is removed by the phaco tip, consecutive drillings into the endonucleus are performed with the phaco tip bevel down. The initial drilling is made from the capsulorhexis edge beneath the main incision, followed by the next consecutive 2–3 drillings towards the center of the nucleus. At the last drilling, the nucleus is deeply impaled and firmly engaged with high vacuum. Then the chopper is inserted under the capsule through the side-port and placed opposite the main incision at the edge of the nucleus. Simultaneously, the chopper is moved from the nucleus periphery toward the phaco tip horizontally to split the nucleus. Both the phaco tip and chopper are then spread apart laterally as they come very close to divide the nucleus into halves. When leathery posterior strands kept nuclear fragments attached to each other, the chopper and phaco tip are reposition at the bottom of the crack to break the strands and propagate a complete division across the full thickness of the entire nucleus. (MP4 11750 kb)
Additional file 2:Video 2. shows the application of consecutive drilling combined with phaco chop technique on Alcon Infiniti Intrepid phacoemulsification system (Alcon Laboratories, Inc., Fort Worth, TX, USA), in which a mini-flared 0.9 mm 30-degree Kelman tip with torsional mode is used through a microincision of 2.2 mm. After the superficial cortex is removed by the phaco tip, consecutive drillings into the endonucleus are performed with the phaco tip bevel down. The initial drilling is made from the capsulorhexis edge beneath the main incision, followed by the next consecutive 2–3 drillings towards the center of the nucleus. At the last drilling, the nucleus is deeply impaled and firmly engaged with high vacuum. Then the chopper is inserted under the capsule through the side-port and placed opposite the main incision at the edge of the nucleus. Simultaneously, the chopper is moved from the nucleus periphery toward the phaco tip horizontally to split the nucleus. Both the phaco tip and chopper are then spread apart laterally as they come very close to divide the nucleus into halves. When leathery posterior strands kept nuclear fragments attached to each other, the chopper and phaco tip are reposition at the bottom of the crack to break the strands and propagate a complete division across the full thickness of the entire nucleus. (MP4 13803 kb)


### Patients

The consecutive drilling combined with phaco chop technique has been adopted in 80 eyes of 65 patients with cataract harder than nuclear opalescence 5 on the Lens Opacities Classification System III scale or mature white cataract with a hard nucleus in the past 12 months. All patients signed an informed consent for participation in this study. All patients were treated in accordance with compliance guidelines outlined by the Declaration of Helsinki.

## Results

In all cases, full thickness segmentation of the hard nuclear including the posterior plate was achieved with this consecutive drilling combined with phaco chop technique. Phacoemulsification and intracapsular implantation of intraocular lens was safely performed in each case. No intraoperative complication such as iris injury, anterior capsule tears, zonulysis or posterior capsule rupture with vitreous loss occurred during surgery. No postoperative complication such as fibrin formation, synechias, severe endothelial cell loss, or endophthalmitis was observed in any case at 6 months postoperatively.

## Discussion

Complete division of nuclei is an essential step to accomplishing uneventful phacoemulsification for hard cataracts. However, the complete disassembly of a hard nucleus is always challenging because the nucleus fibers are strong and densely packed. In addition, hard cataract is commonly complicated by the paucity of protective cortex and epinuclear layer, the laxity of the zonules, and the fragility of the capsule. All these factors may increase the risk of injury to the supportive structures of the lens during phacoemulsification.

Many techniques have been introduced to achieve nuclear fragmentation, such as phaco chop originally introduced by Nagahara [3], divide and conquer described by Gimbel [4], and stop and chop made popular by Koch and Katzen [5]. There are also variable modified surgical techniques claimed to enhance the efficacy of hard nucleus division [6–11]. These surgical techniques frequently end up in a longer time of high intensity compressive phaco forces and extensive manipulation. Multiple maneuvers such as sculpting, incising, crushing, or drilling are adopted to create a groove or trench for the initial chopping and further segmentation of the rock-hard nucleus. The stressful mechanics of fracture to break the dense nucleus, quite often do not succeed, resulting in incomplete nuclear segmentation and intact posterior plate.

Among these techniques, phaco chop may have advantages of being fast and effective for coaxial MICS in hard cataract [12]. In the original phaco chop technique, the phaco tip is buried in the center of the epinucleus with high vacuum, the phaco chopper is then positioned at the edge of the nucleus opposite the main incision and pulled toward the phaco tip horizontally. The two instruments are then spread apart laterally to divide the nucleus into halves. However, due to the physically robust nature of the dense nucleus and limited manipulation of the two instruments in region close to the main incision, the nucleus in this region quite often remain mechanically intact after the initial chop, resulting in incomplete nuclear segmentation. Surgeons have to rotate the nucleus to position the unchopped region opposite to the phaco tip and subsequent repeated chops are performed to achieve a complete division of the nucleus. Some techniques such as phaco-flip may be beneficial in cases in which the first chop is not successful [13]. However, significant force is applied to the zonules and lens capsule when additional manipulation is needed, which may not be suitable in cases with weak zonules or fragile capsule.

To overcome the drawback of the phaco chop for coaxial MICS in hard cataract, we describe a technique that facilitates complete division of dense nucleus. We adopted the consecutive drilling idea from mining engineering [14]. Miners drill multiple holes in a line and then use jimmy to exert inside-out forces to crack a rock into halves. Normally brittle hard materials are stronger in compression but weaker in tensile strength according to the Griffiths theory of brittle fracture [15]. So it is always easier to segment these hard objects with inside-out dispersive mechanical forces than with compressive forces. The dense bulky central nucleus of a hard cataract is even harder and unbreakable. The authors first perform a series of deep drilling into the endonucleus to disintegrate the mechanical structure of the dense bulky central nucleus. The weakening of the hardest central core will make the fracture line to propagate posteriorly easily during the following action of phaco chop. The consecutive drilling also creates a deep trench close to the incision side, which facilitates the further full segmentation by the dispersive force from the opposite lateral motion of phaco tip and chopper. In addition, consecutive drilling debulks the central portion of the nucleus, which allows safer and easier endocapsular phacoemulsifcation of the nuclear fragments after chopping or splitting.

The consecutive drilling combined with phaco chop technique has several advantages over conventional nuclear disassembly. First, it is highly efficient in full thickness segmentation of dense nucleus with the first chop because the mechanics structure of central nucleus is weakened by consecutive drilling before phaco chop. Second, the technique has high safety since it applies minimal force to the zonules and lens capsule. In addition, it can minimize unwanted ultrasound energy to the corneal endothelium because the phaco tip is placed bevel down and fully buried into the nucleus when ultrasound energy is delivered for drilling and impaling. This is particularly important when patients have a potential for weak zonular fibers and low endothelial cell counts. Third, the technique is minimally invasive and can be carried out through microincision down to 1.8 mm with relatively high efficacy. Forth, the technique can be easily mastered even by beginners and does not have a steep learning curve.

The following should be considered carefully when using this technique. An approximately 6.0 mm capsulorhexis should be created. This adequately large CCC diameter will allow the initial drilling to be performed as peripheral as possible without breaking the edge of the capsulorhexis. Surgeons should not push the nucleus with the phaco tip but instead use high vacuum and continuous ultrasound energy, which will prevent zonular stress. As the lens thickness increases from periphery to the center, the depth of consecutive drilling increases gradually. Surgeons can estimate the drilling depth by the length of phaco tip exposed from the silicone sleeve. Although this technique can be done with the phaco tip “bevel down” or “bevel up” depending on the surgeon’s preference, “bevel down” style is recommended when performing the consecutive drilling. First, “bevel down” is more efficient because the tip is fully obstructed with high vacuum and almost all the ultrasound energy is delivered into the nuclear when drilling. Second, “bevel down” may be safer because the potential damage of cavitation effect of ultrasound to the endothelium is minimized when the bevel is facing away from the endothelium.

## Conclusions

We have described a modification of the original phaco chop technique named “consecutive drilling combined with phaco chop” in coaxial microincisional phacoemulsifcation. To the best of our knowledge, this technique has not been described in the literature previously. The technique is an efficient, safe, simple, and swift procedure for full-thickness nuclear segmentation, delivering advantage of microincisional phacoemulsifcation for hard cataract with fewer ocular complications. Further clinical studies are needed to objectively compare this technique with other techniques for managing hard cataracts.
